# Retraction: Concordance and characterization of massively parallel sequencing at 58 STRs in a Tibetan population

**DOI:** 10.1002/mgg3.2383

**Published:** 2024-02-12

## Abstract

Li H., Zhang C., Song G., Ma K., Cao Y., Zhao X., Yang Q., Xie J. (2021). Concordance and characterization of massively parallel sequencing at 58 STRs in a Tibetan population. *Molecular Genetics & Genomic Medicine*, 9(4), e1626. https://doi.org/10.1002/mgg3.1626.

The above article, published online on 25 February 2021 in Wiley Online Library (wileyonlinelibrary.com), has been retracted by agreement between the journal Editor in Chief, Suzanne Hart, and Wiley Periodicals LLC. Following publication of this article, concerns were raised by a third party regarding consent and ethical approval for the research undertaken and reported in the article. The publisher and representatives of the journal's editorial board undertook a review of the consent documentation provided, against the research performed and reported in the article. The review uncovered inconsistencies between the consent documentation and the research reported; the documentation was not sufficiently detailed to resolve the concerns raised. As a result, the parties have made the decision to retract the article. In addition, consent documentation did not give approval for data associated with this article to be shared publicly. The associated data for this article has been withdrawn from the publication record following a concern that the data could be used to identify those who have participated in the research. Jianhui Xie stated on behalf of all co‐authors that they do not agree to this retraction.

Li H., Zhang C., Song G., Ma K., Cao Y., Zhao X., Yang Q., Xie J. (2021). Concordance and characterization of massively parallel sequencing at 58 STRs in a Tibetan population. *Molecular Genetics & Genomic Medicine*, 9(4), e1626. https://doi.org/10.1002/mgg3.1626.

The above article, published online on 25 February 2021 in Wiley Online Library (wileyonlinelibrary.com), has been retracted by agreement between the journal Editor in Chief, Suzanne Hart, and Wiley Periodicals LLC. Following publication of this article, concerns were raised by a third party regarding consent and ethical approval for the research undertaken and reported in the article. The publisher and representatives of the journal's editorial board undertook a review of the consent documentation provided, against the research performed and reported in the article. The review uncovered inconsistencies between the consent documentation and the research reported; the documentation was not sufficiently detailed to resolve the concerns raised. As a result, the parties have made the decision to retract the article. In addition, consent documentation did not give approval for data associated with this article to be shared publicly. The associated data for this article has been withdrawn from the publication record following a concern that the data could be used to identify those who have participated in the research. Jianhui Xie stated on behalf of all co‐authors that they do not agree to this retraction.

